# Implementing a Need-Adapted Stepped-Care Model for Mental Health of Refugees: Preliminary Data of the State-Funded Project “RefuKey”

**DOI:** 10.3389/fpsyt.2019.00688

**Published:** 2019-09-27

**Authors:** Beata Trilesnik, Umut Altunoz, Janina Wesolowski, Leonard Eckhoff, Ibrahim Ozkan, Karin Loos, Gisela Penteker, Iris Tatjana Graef-Calliess

**Affiliations:** ^1^Department of Psychology, Humboldt University of Berlin, Berlin, Germany; ^2^Department of General Psychiatry and Psychotherapy, KRH Psychiatry Wunstorf, Wunstorf, Germany; ^3^Department of Economic and Social Psychology, Institute of Psychology, Georg-August-University Goettingen, Goettingen, Germany; ^4^Faculty of Physics, Ludwig Maximilian University of Munich, Munich, Germany; ^5^Psychiatric Outpatient Clinic, Asklepios Fachklinikum Göttingen, Göttingen, Germany; ^6^NTFN e.V., Hannover, Germany; ^7^Department of Psychiatry, Social Psychiatry and Psychotherapy, Hannover Medical School, Hannover, Germany

**Keywords:** mental health, post-migration living difficulties, refugees, stepped-care model, intercultural opening

## Abstract

**Introduction:** Refugees have been shown to be a rather vulnerable population with increased psychiatric morbidity and lack of access to adequate mental health care. By expanding regional psychosocial and psychiatric-psychotherapeutic care structures and adapting psychiatric routine care to refugees’ needs, the state-funded project “refuKey” based in Lower Saxony, Germany, pursues to ease access to mental health care and increase service quality for refugees. A stepped-care treatment model along with intercultural opening of mental health care services is proposed.

**Methods:** The project is subject to a four-part evaluation study. The first part investigates the state of psychiatric routine care for refugees in Lower Saxony by requesting data from all psychiatric clinics, participating and non-participating ones, regarding the numbers of refugee patients, their diagnoses, settings of treatment, etc. The second part explores experiences and work satisfaction of mental health care professionals treating refugees in refuKey cooperation clinics. The third part consists of interviews and focus group discussions with experts regarding challenges in mental health care of refugees and expectations for improvement through refuKey. The fourth part compares mental health parameters like depression, anxiety, traumatization, somatization, psychoticism, quality of life, as well as “pathways-to-care” of refuKey-treated refugees before and after treatment and, in a follow-up, to a non-refuKey-treated refugee control group.

**Results:** RefuKey-treated refugees reported many mental health problems and estimated their mental health burden as high. The symptoms decreased significantly over the course of treatment. Mental health in the refuKey sample was strongly linked to post-migration stressors.

**Discussion:** The state of mental health care for refugees is discussed. Implications for the improvement and the need for adaptation of routine mental health care services are drawn.

## Introduction

Recent years have marked a stark increase in refugees^1^ leaving their home countries due to conflicts, poverty, and persecution. The numbers for forced displacement are highest on record with 28.5 million globally displaced people in 2018 (1). In 2015, roughly 890,000 asylum seekers arrived in Germany, of whom 441,900 officially applied for asylum, making Germany the most popular host country in the EU (2).

Several studies have shown an elevated risk for mental disorders in samples of refugees (3–5). A recent meta-analysis on refugee mental health reported increased prevalence rates for posttraumatic stress disorder (PTSD; 4.4–86%), depression (2.3–80%), and anxiety disorders (20.3–88%) (3). Compared to studies with small sample sizes, studies of higher methodological quality with large sample sizes generally report lower prevalence rates of mental disorders in refugees (3, 4). Even though prevalence rates should be interpreted with caution due to the heterogeneity of study designs and sample characteristics, there is evidence that refugees constitute a particularly vulnerable population with about 10 times increased risk of PTSD in comparison to the host countries’ native population [e.g., Refs. (3, 4)]. Furthermore, there is a growing body of evidence that the incidence of psychosis in migrant populations is elevated compared to non-migrant population (6). Recent studies further report that risk of developing psychotic disorders is higher for refugees than non-refugee migrants (7, 8).

In this context, research highlights the importance of post migratory factors showing that various stressors related to socioeconomic difficulties in host countries, social and interpersonal challenges, lengthy asylum-seeking process, and complicated immigration policies facilitate long-term mental health problems in refugees after resettlement (3, 9–11). For instance, Knipscheer et al. (12) reported negative effects of the asylum-seeking process on mental health, as they found an association between lack of refugee status and symptom severity of PTSD and depression. Generally, stressful life events that frequently moderate the onset of psychotic disorders might be particularly common among refugees (e.g., migration itself, physical and sexual abuse, perceived discrimination) (9, 13, 14).

Despite the fact that refugees, due to various risk factors, represent a population vulnerable to mental disorders, several structural and social barriers in host countries are preventing this population from receiving adequate mental health care (15). For one, refugees report difficulties in dealing with a foreign, complex health care system (16). Language deficiency represents one of the major access barriers to and a potential source of miscommunication within the health care system, leading to a risk of inadequate clinical assessment (15, 17). Further, the cultural background of an individual might affect symptom patterns, perception of mental disorders, as well as beliefs about mental health care and acceptability of certain treatments (15, 18). Therefore, lack of intercultural competence of mental health care providers, along with the inability to understand the effect of culture on different aspects of mental disorders, present an impediment for adequate health care (19,).

In many countries, including Germany, asylum seekers’ access to health care is limited directly by law. For the first 15 months after arrival in Germany, asylum seekers’ access to health care only covers measures deemed essential for life preservation [e.g., emergency medical care, treatment for acute and painful conditions, care during pregnancy and childbirth, vaccinations; AsylBLG^2^ sections 4 and 6 (21)]. During this period, regular treatment of mental disorders is not covered. Since 2016, asylum applications are steadily decreasing in Germany (2). However, the current situation still is a great challenge on how to address the needs of refugees for mental health care adequately. In that regard, stepped-care models may provide a promising framework, as they have proven their effectiveness for various disorders in different settings and regions of the world and are recommended for routine care practices (22). Within stepped care, treatment is distributed to several steps with different treatment thresholds, ranging from lower to higher intensities. This enables an optimized provision of mental health care, which takes into account limited clinical and therapeutic resources (22).

The refuKey project aims to enhance regional psychosocial, psychiatric, and psychotherapeutic care services for refugees by means of stepped-care approaches optimizing regular mental health care in Lower Saxony, Germany (23). Within the scope of the refuKey project, psychosocial counseling centers (PCCs) have been founded close to refugee reception centres and joined forces with a psychiatric clinic nearby as cooperating competence centres. The project provides the clinics and PCCs with professional interpreters and academic refuKey staff to support treatment teams in coping with bureaucratic procedures as well as to help reduce diagnostic and therapeutic insecurities in dealing with refugee mental health and to ensure optimal regional networking. The refuKey staff is composed of clinical psychologists, psychiatrists, psychotherapists, and social workers who are trained and experienced in transcultural competence. By integrating low- and high-threshold programs, the refuKey project works to provide need-adapted mental health care for refugees and to promote intercultural opening of the mental health care system. The project started in May 2017 and is a cooperation between the Network for Traumatized Refugees in Lower Saxony (NTFN e.V.) and the German Association for Psychiatry, Psychotherapy, and Psychosomatics (DGPPN). The project is funded by the Ministry of Social Affairs, Health and Equality of Lower Saxony. RefuKey is meant to serve as a model/pilot project for further action. This paper aims to present the first naturalistic data of the refuKey project evaluation study.

### Objectives

The study’s objective is to evaluate the efficacy of the project by answering the following three questions: First, do refugees have better access to mental health care as a result of the project? Better access is defined by an increase in the number of refugee patients treated in participating psychiatric clinics and PCCs as well as an increase in referrals between these institutions. Second, is there a decrease in re-hospitalization rates of refugees in participating psychiatric clinics, as well as a significant improvement of the mental state of refuKey-treated refugees as compared to non-refuKey-treated refugees? Third, is there a decrease of work-related strain in mental health care professionals working with refugees over the course of the project in participating psychiatric clinics?

## Methods

### Study Design

**Figure 1:**
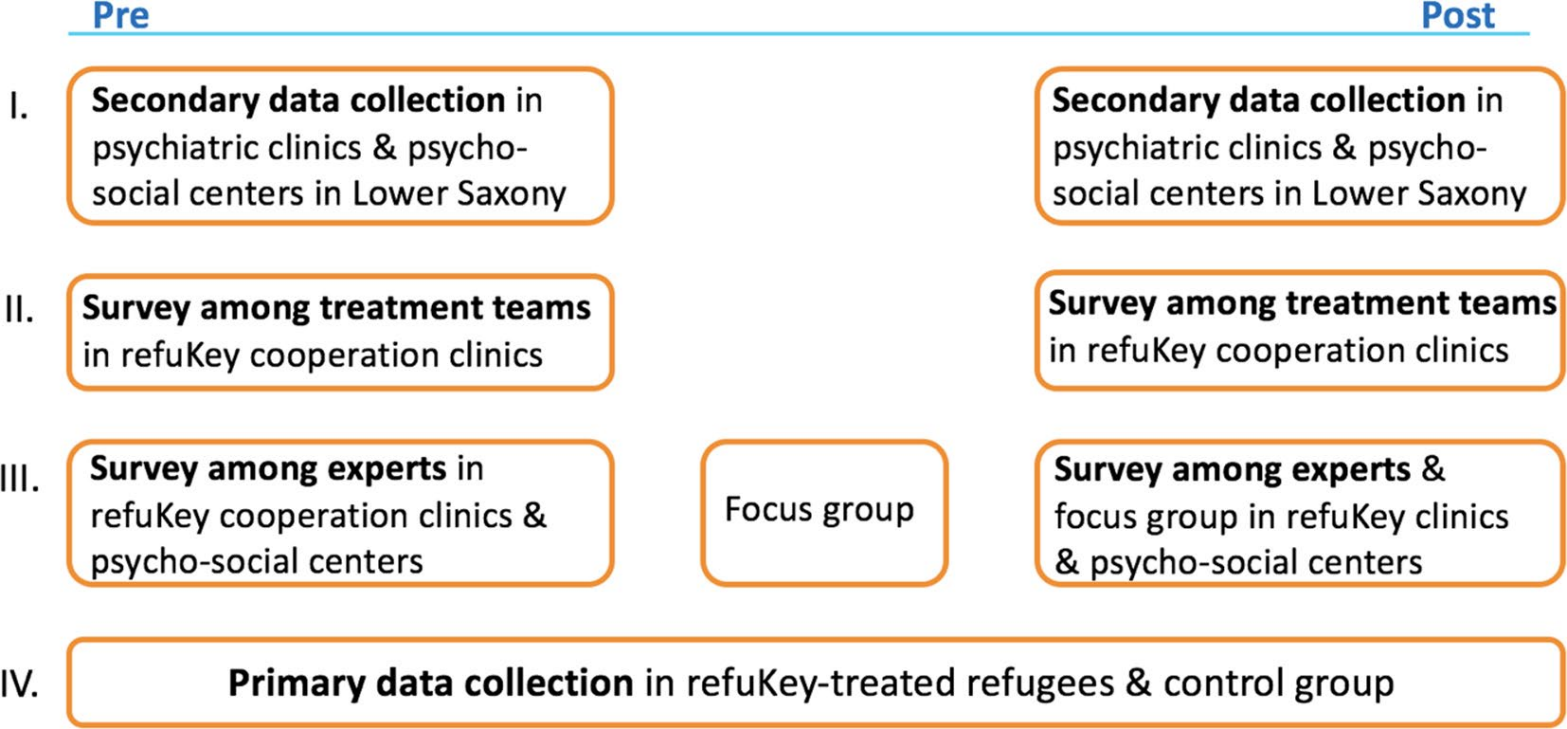
RefuKey evaluation study design.

The study uses a complex naturalistic mixed-methods multi-centric design examining different aspects of treatment in four study parts (**Figure 1**). The first part investigates the state of psychiatric routine care for refugees in Lower Saxony at the start and end of the project. For this, secondary data from all psychiatric clinics in the state, both participating and not participating in refuKey, were requested *via* an online survey. The aim of the secondary data collection is to measure whether our project can facilitate access to standard psychiatric routine care comparing patient numbers, re-hospitalization rates, and referrals to follow-up treatment between refuKey participating and non-participating clinics. The second part explores the experiences and work-related strain of mental health care professionals involved in mental health care provision for refugees in psychiatric clinics participating in refuKey as well as in a non-participating clinic as a control group, is collected using standardized questionnaires provided at the start and end of the project. Challenges in providing mental health care to refugees and expectations for improvement through refuKey are assessed throughout the project in the third part with structured interviews and focus group discussions. The fourth and main part of the study measures mental health as well as “pathways-to-care” of refuKey-treated refugees before and after treatment using a standardized questionnaire to examine if refuKey treatment is effective. RefuKey treatment outcomes will be compared to a non-refuKey-treated refugee control group to check if refuKey treatment is superior to treatment as usual.

The study was approved by the Ethics Committees of Hannover Medical School and the Medical Association of Lower Saxony.

### Sample

For the first study part, chief physicians of psychiatric clinics were recruited *via* a presentation of the project to the Committee^3^ of chief physicians of all psychiatric clinics in Lower Saxony and by sending a link to the survey through the committee’s mailing list. Seven clinics participated in the survey.

The sample of the ongoing second study part includes professionals who treat and care for refugee patients in psychiatric clinics participating in refuKey. We ask 10 representatives of a multi-professional team *per* psychiatric setting covering the professions of psychiatrists; psychologists; occupational, art, music, and body therapists; nurses; and social workers where applicable according to specific settings. A sample of 100 psychiatric clinic employees is planned. All participants are informed about the study by an information sheet and sign the form of consent prior to participation.

In this study part, an expert is defined as someone who works in refugee mental health provision at different levels of delivery within institutions participating in refuKey. The expert sample consists of members of the project steering group, directors of psycho-social centers, clinic directors, clinic personnel, as well as refuKey personnel and counts 14 participants at the start of the project.

Refugees receiving treatment and counseling in refuKey PCCs or psychiatric clinics are included in the sample of the fourth study part. Data collection for this part of the study will continue until the end of the project. Data are collected in four psychiatric clinics (Asklepios Clinic for Psychiatry and Psychotherapy in Goettingen, AWO Psychiatric Centre in Koenigsluther, Karl-Jaspers-Clinic in Oldenburg, Niels-Stensen-Clinics in Bramsche) and five PCCs (Brunswick, Goettingen, Lueneburg, Oldenburg, Osnabrueck) in Lower Saxony, Germany. Written informed consent is obtained from all participants. All participants are informed about the study by an information sheet and sign the form of consent prior to participation.

**Figure 2:**
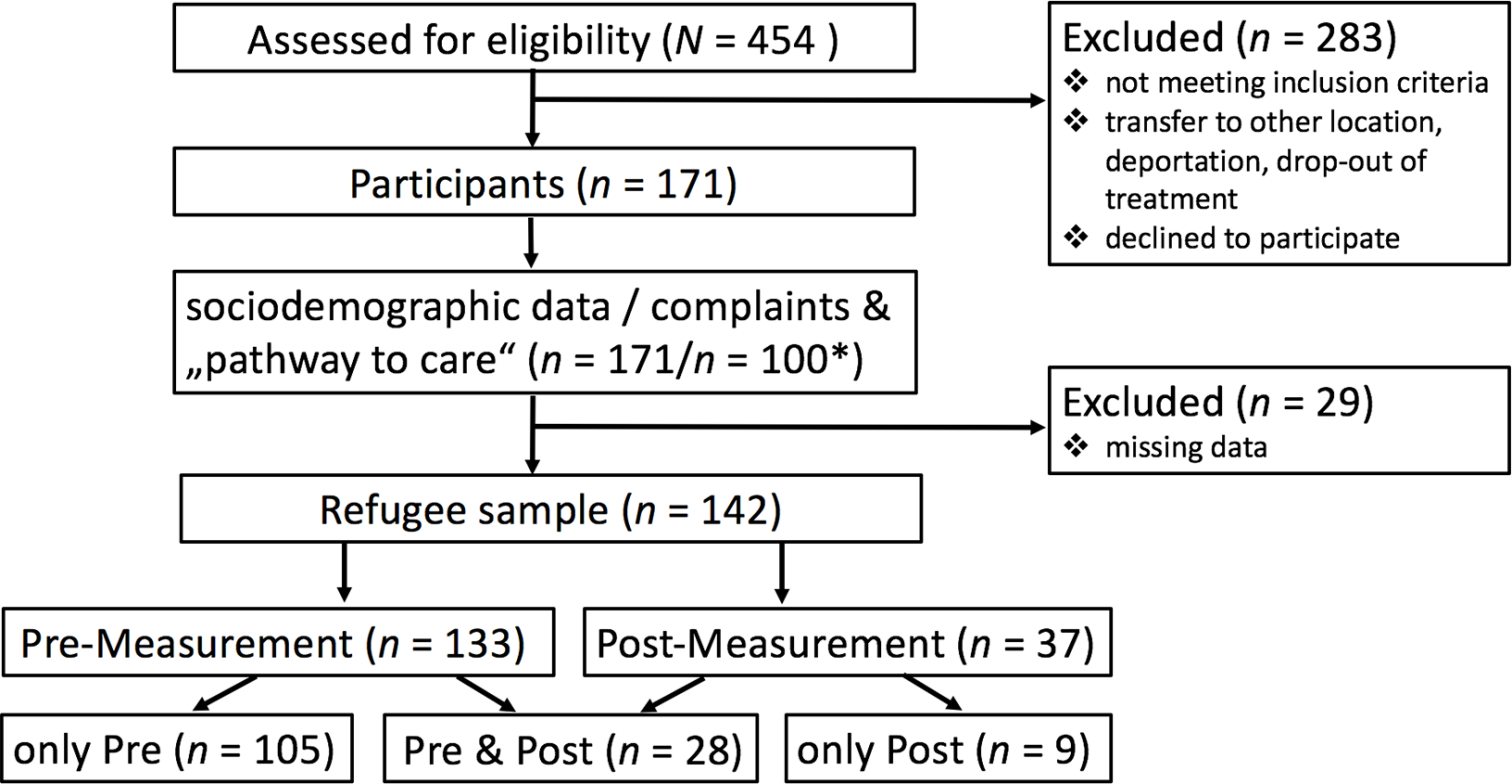
Refugee sample. *Due to the naturalistic design of the study and the hardships of data collection along the treatment process, the information about complaints and “pathway to care” is missing for 71 participants.

Refugees who make use of refuKey open counseling hours irregularly as well as refugees who are not capable of filling out a questionnaire due to insufficient educational background or acute mental health conditions (e.g., acute suicidality or acute psychotic symptoms) are excluded from the sample. So far, 171 refugees have participated in the study, while 283 refugees were excluded due to referral to a different mental health institution, transfer to another city, deportation, refusal to participate in the study, or meeting the exclusion criteria. A total of 133 pre-treatment as well as 28 post-treatment measurements including 28 paired pre- and post-treatment measurements were included in the analyses, while 29 participants were excluded due to missing data (**Figure 2**). Additional information about current symptoms and complaints as well as their severity was available for only 100 of the participants. Data from the control group will be collected as a double-blind study from all refugee patients treated in a psychiatric clinic in Lower Saxony, which does not participate in refuKey at a future point in time.

### Measures

#### Secondary Data Collection

In the first part of the study, secondary data were collected in psychiatric clinics in Lower Saxony *via* a short online survey consisting of 11 questions. The survey asked for numbers of refugee patients treated, their diagnoses and reasons for admission, type of diagnostic assessment, settings, types and length of treatment, use of interpreters, re-hospitalization rate, referral to follow-up treatment in the first quarter of 2018, and for a rating of suggested impediments to the quality of mental health care for refugees.

#### Survey Among Treatment Teams

In the second part, we applied the Maslach Burnout Inventory— Human Services Survey (MBI) by Maslach and Jackson (24) to measure the work-related strain in the care for refugee patients. The MBI consists of 21 items rated from 1 to 6 (1 = “never” to 6 = “very often”). Internal consistency was reported to lie between Cronbach’s α of .71 and .82 (24).

We used the Current Mood Scale (Aktuelle Stimmungsskala, ASTS) by Dalbert (25) to assess personnel’s mood while providing mental health care for refugee and non-refugee patients. The ASTS assesses mood in the categories of sadness, hopelessness, tiredness, anger and positive mood with 19 items on a seven-point Likert scale (1 = “extremely” to 7 = “not at all”). The internal consistency is relatively high (Cronbach’s α = .83–.94) (25).

Additionally, sociodemographic data, such as age, sex, migration background, and occupation, as well as workplace-related data, such as work setting, income, and whether or not refugees are treated, are collected.

#### Survey Among Experts

The experts in the third part of the study were interviewed using a structured questionnaire composed of open questions asking about challenges in the mental health care of refugees. In particular, they were asked about financial constraints, networks and cooperation, difficulties for treatment teams, and barriers to mental health care access faced by refugees. Expectations about improvements through refuKey were asked about as well.

#### Primary Data Collection

A standardized questionnaire consisting of validated scales widely used in international refugee research is applied in the fourth part of the study (Health TA Center: Assessment for Trauma and Mental Health in Refugees, www.refugeehealthta.org). The questionnaire is available in eight languages: German, English, Arabic, Farsi, Dari, French, Serbian, and Russian. It contains, if available, validated adaptations of the scales in those languages and, if not, translations into them and back. The questionnaire was tested in a test run.

##### Mental Health

We assessed the following mental health parameters: general mental well-being with the Warwick Edinburgh Mental Well-Being Scale by Tennant et al. (26), depression and anxiety with the Hopkins Symptom Checklist 25 by Derogatis et al. (27), somatization and psychoticism with the Symptom Checklist 90 by Derogatis (28), traumatization with the Harvard Trauma Questionnaire by Mollica et al. (29), as well as quality of life with the WHO Quality of Life Questionnaire (30).

The Warwick Edinburgh Mental Well-Being Scale (WEMWBS) measures general mental well-being as a sum score of 14 positively worded items rated on a five-point Likert scale. The questionnaire shows high internal consistency (α = .91) (26).

The Hopkins Symptom Checklist 25 (HSCL-25) is a well-known and widely used clinical screening instrument. It assesses anxiety experienced in the last 7 days by means of 10 items and depression by means of 15 items with four categories of response (1 = “not at all,” 2 = “a little,” 3 = “quite a bit,” 4 = “extremely”). Internal consistency is high (α = .89) (31). The scale has been adapted to many languages and has been used with a number of refugee groups.

The Symptom Checklist 90-R (SCL-90) evaluates a broad range of psychological symptoms experienced in the last 7 days rated on a five-point Likert scale. Internal consistency was shown to be at α = .77 for the somatization subscale and α = .73 for the psychoticism subscale (32).

The Harvard Trauma Questionnaire (HTQ) is a cross-cultural screening instrument assessing trauma exposure and its subjective description as well as head trauma and trauma-related symptoms in refugees. In this study, only the last part composed of 30 items regarding posttraumatic symptoms experienced in the last week was administered. The first 16 items reflect the DSM-IV criteria for PTBS and the other 14 items describe trauma-related symptoms. Items are rated on a 1 (“not at all”) to 4 (“extremely”) Likert scale. Internal consistency of the scale is high (α = .96) (29).

The WHO Quality of Life Questionnaire (WHOQoL)—BREF is a short version of the WHO Quality of Life scale, which is cross-culturally applicable. It has 26 items and a response scale ranging from 1 to 5. The questionnaire assesses physical and psychological quality of life, social relationships, and environment. Psychometric properties were tested internationally and have been reported as high (α = .78, α = .89, α = .70, and α = .80, respectively) (33).

##### Other Factors

Furthermore, we measured current migrant life stressors with the Post-Migration Living Difficulties Checklist (PMLDC) by Silove et al. (34). The 17-item version that we selected is rated on a scale from 1 (“no problem at all”) to 5 (“a very serious problem”) and showed good internal consistency of α = .72 (35). Additionally, the three-item scale for perceived discrimination by Finch et al. (36) was used and adapted to the refugee context (internal consistency: α = .76). The sociodemographic and flight-specific data as well as data about current complaints and help-seeking behavior are assessed with the National Migration Questionnaire by the German Association for Psychiatry, Psychotherapy, and Psychosomatics.

### Statistical Analysis

Statistical analyses of the data collected so far were conducted using IBM SPSS Statistics 24 (IBM-Deutschland GmbH, Munich, Germany). Descriptive statistics are given in terms of means and standard deviations for continuous variables, and counts and percentages for categorical variables. Prevalence rates were calculated on the basis of cutoff scores, where applicable. Comparability of the participant and non-participant sample was assessed with analyses of variance (ANOVAs) regarding continuous variables, and with chi-square-analysis (χ^2^) regarding discrete variables. Descriptive analyses were followed by multivariate analyses of variance (MANOVAs), after having performed power analysis^4^, comparing the mental state of refugees treated in psychiatric clinics vs. PCC, and by a paired samples *t* test for a comparison of the refugees’ mental state before and after treatment. Analyses were conducted at a significance level of *p* = .05. Assumption of normal distribution was tested in the paired sample using the Shapiro–Wilk Test. The data were normally distributed except for psychoticism, somatization, and trauma symptomatic in the post-treatment measurement. In these cases, the Wilcoxon Signed Rank Test was applied in addition to the paired samples *t* test. Finally, Pearson’s correlation analysis was carried out for the link between post-migration living difficulties and mental health indices.

## Results

### Secondary Data Collection

The rate of return in this part of the study was relatively low. Of the 32 psychiatric clinics that were approached in the state of Lower Saxony, only 7 completed the survey. Lack of available data about refugee patients in the hospital documentation systems was the major reason for non-participation.

**Figure 3:**
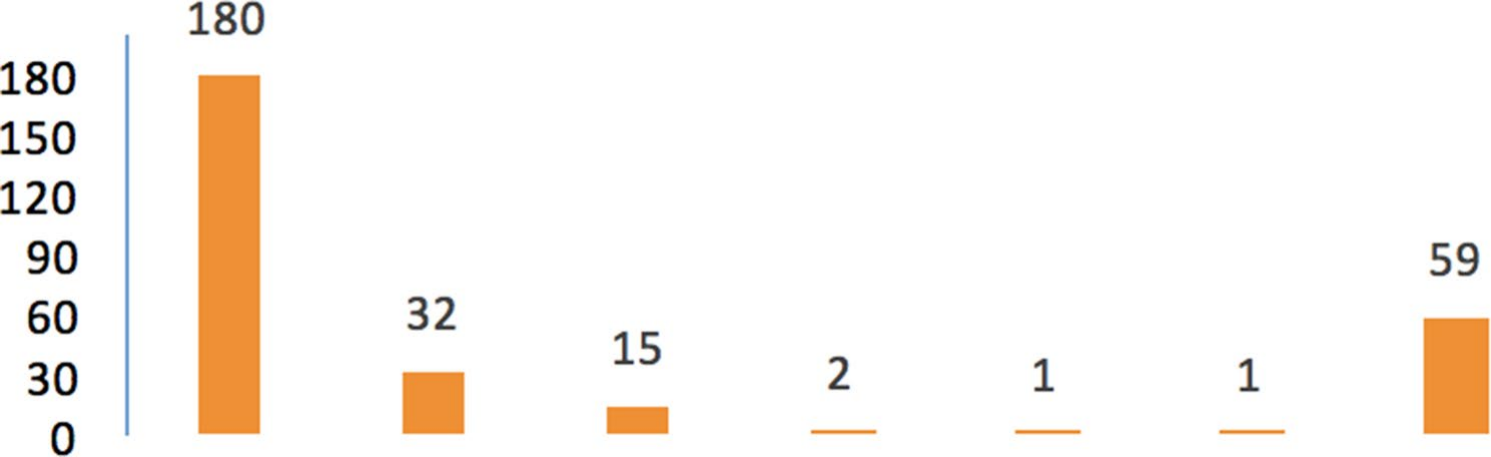
Numbers of refugee patients in seven psychiatric clinics in Lower Saxony in the first quarter of 2018 (*N* = 7 psychiatric clinics).

The clinics that did complete the survey reported varying numbers of refugee patients across settings, ranging from 1 to 180 patients in the first quarter of 2018 (see **Figure 3**).

The chief physicians of these clinics (*N* = 5) also rated a given variety of impediments to high-quality mental health care for refugees, i.e., differences in gender roles, institutional racism, time consumption, lack of compliance, lack of trust by the patient, different systems of values, different presentation of symptoms, different understanding of disease, diagnostic uncertainty, and linguistic difficulties. Most notably, out of these, language barriers and lack of sufficient time were reported by all clinics as significant impeding factors. The answers to the other questions in this survey are omitted in this report because of the low return rate.

### Survey Among Experts

**Figure 4:**
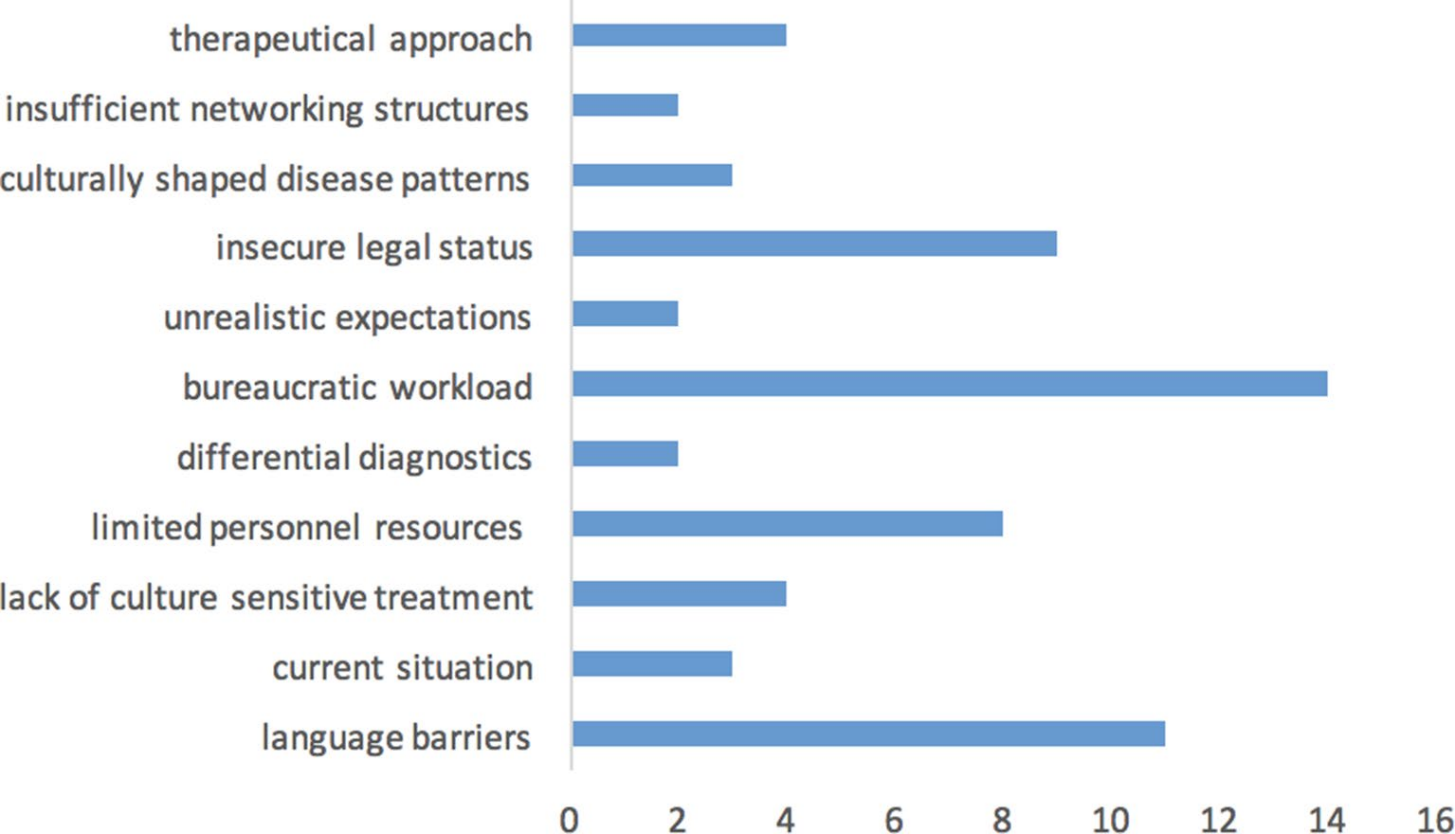
Impediments to high quality of mental health care for refugees and the number of namings (*N* = 14 experts).

The impediments to high-quality mental health care for refugees reported by the expert sample match to some extent with those reported by the clinics (see **Figure 4**). In particular, language barriers and limited personnel resources were among the four most widely reported factors together with the insecure legal status of refugees and bureaucratic workload, which was the most-reported factor of all. The other results of the expert survey fall outside the scope of this article.

### Primary Data Collection

Sociodemographic and flight-specific data reported by the refugees treated or counseled within refuKey-participating institutions are presented in **Table 1**. The participant sample in this study so far consisted of 54% males and 46% females, with a mean age of 31.07 years. Participants came from 30 different countries of origin, with Afghanistan (15.4%), Iran (14.2%), Syria (8.0%), and Iraq (6.8%) ranking at the top, followed by Kosovo, Lebanon, Turkey, and Sudan (3.1% each). Notably, over 60% of the participants had an insecure residency status (ranging from threat of deportation to having a residence acceptance), while almost one third of participants did not or could not report any information on residence status.

**Table 1:** Sociodemographic and flight-specific characteristics for participating and non-participating refugee patients (ANOVA and chi-squared analysis).

	Total sample (*N* = 454)			Participants (*N* = 171)			Non-participants (*N* = 283)			*F/*χ^2^	*df*	*p*
	*M/N*	*SD*/%	Range	*M/N*	*SD*/%	Range	*M/N*	*SD*/%	Range			
Age	31.6	(10.5)	16–67	31.1	(9.8)	17–62	31.9	(10.8)	16–67	.639		n.s.
Gender												
Male	243	59%		74	54%		169	61%		1.969	1	n.s.
Female	170	41%		63	46%		107	39%				
No information	41			34			7					
Marital status												
Married/in partnership	157	41.2%		54	41.9%		103	40.9%		.523	4	n.s.
Single/divorced/widowed	224	58.8%		75	58.1%		149	59.1%				
No information	73			42			31					
Education												
Illiteracy	16	9.1%		9	9%		7	9.3%		19.061	6	<.01
No school education	37	21.4%		22	22%		15	20%				
Secondary education	19	10.8%		15	15%		4	5.3%				
Occupational training	4	2.3%		4	4%		0	0%				
High school diploma	24	13.7%		19	19%		5	6.7%				
University	29	16.6%		17	17%		12	16%				
No information	46	26.3%		14	11%		32	42.7%				
Legal status												
Threat of deportation	49	10.8%		20	11.7%		29	10.2%		14.922	7	<.05
Temporary toleration	49	10.8%		21	12.3%		28	9.9%				
Residence acceptance	177	39.0%		56	32.7%		121	42.7%				
Other	16	3.5%		8	4.7%		8	2.8%				
Residence permit	37	8.1%		9	5.3%		28	9.9%		7.324	6	n.s.
Permanent residence permit	2	.4%		1	.6%		1	.3%				
Visa	7	1.5%		2	1.2%		5	1.8%				
No information	117	25.7%		54	31.6%		63	22.3%				
Duration of residence	27.2	(33.9)	1–351	30.6	(30.1)	1–267	25.2	(35.9)	5–351	1.967		n.s.
Reasons for flight												
War	64	38.1%		39	40.2%		25	35.2%		.441	2	n.s.
Natural disaster	3	1.8%		2	2.1%		1	1.5%		.145	2	n.s.
Economic deprivation	24	14.4%		15	15.4%		9	12.8%		.263	2	n.s.
Political/religious persecution	77	46.1%		45	46.4%		32	45.7%		.061	2	n.s.
Social reasons	28	16.8%		19	19.6%		9	12.8%		1.332	2	n.s.
Individual reasons	29	17.4%		20	20.6%		9	12.8%		1.715	2	n.s.
Sex-based/sexual persecution	23	13.8%		9	9.3%		14	20%		4.147	2	n.s.
Other	38	22.7%		23	23.7%		15	21.4%		.160	2	n.s.

Participants/non-participants refuKey refugee patients participating/not participating in the evaluation study; for education and reasons for flight, N = 175, N = 100, and N = 75, respectively; as the data were collected at different points during the treatment process, this information is unfortunately missing for the other 71 participants and 208 non-participants; M, mean; SD, standard deviation; p, level of significance; F value on the F; distribution, test statistics in an analysis of variance; *χ*2, test statistics in chi-squared tests; df degrees of freedom; n.s, not significant.

The refugee patients participating in this study are comparable to those not participating or excluded from the study, in terms of age, gender, and marital status as well as duration of residence and reasons for flight. However, we found significant differences in levels of education [χ*^2^*(6) = 19.061, *p* < .01] as study participants were more educated than non-participants. In terms of legal status, fewer study participants had a residence permit and more are under threat of deportation or are only temporarily tolerated in comparison to non-participants [χ^2^(7) = 14.922, *p* < .05].

**Figure 5:**
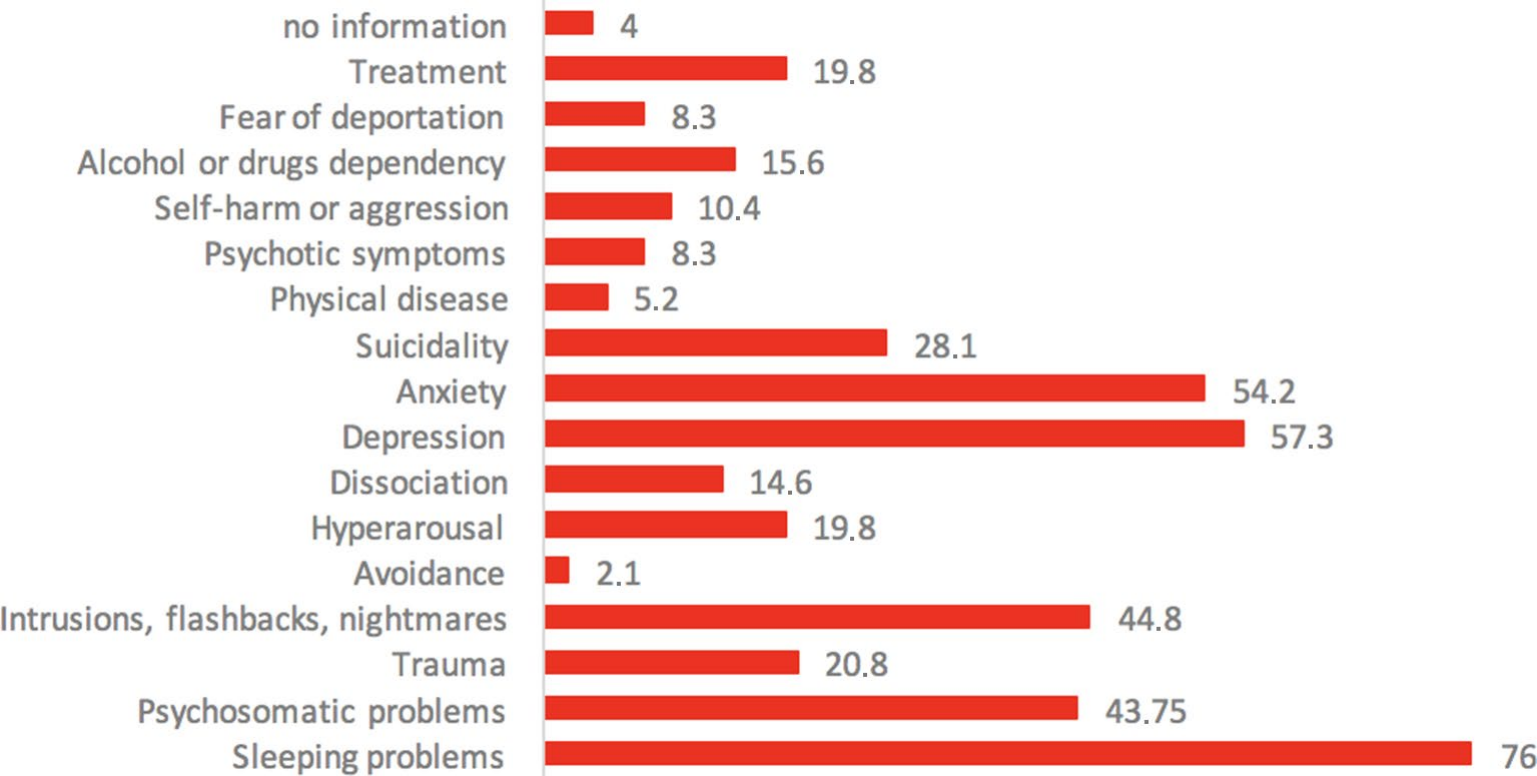
Reported symptoms and complaints in % (*N* = 100 study participants).

Complaints and symptoms reported by the participants as reason for seeking help in refuKey are given in **Figure 5**. By far, the most commonly encountered symptom was that of sleeping problems, with three quarters of participants (76%) suffering from it. Furthermore, approximately half of the participants reported symptoms of depression (57.3%), anxiety (54.2%), psychosomatic problems (43.7%), as well as posttraumatic symptoms (44.8%). The mental health burden of refugees in our sample was very high, reflected by participants’ symptom severity estimation shown in **Table 2**. Three quarters (74.8%) reported strong to extreme symptom severity.

**Table 2:** Estimation of symptom severity/burden in % (*N* = 100 study participants).

No answer	0	1	2	3	4	5	6	7	8	9	10
	No	Very light		Light		Moderate		Strong		Extreme	
12.6	.6	1.1	0	1.1	1.1	3.4	5.2	7.5	14.4	18.4	34.5

The prevalence of clinically relevant symptoms and their severity decreased in the course of treatment as demonstrated in **Table 3**. Prevalence rates decreased from 92.6% to 72.4% for depressive symptoms, from 85.7% to 75.9% for anxiety, from 96.6% to 63% for psychoticism, from 79.3% to 42.9% for somatization, and from 69% to 64.3% for traumatization. Prevalence of very severe symptoms went down to zero after treatment.

**Table 3:** Prevalence of clinically relevant symptoms and their severity before and after treatment.

	Pre-treatment measurement (%)						Post-treatment measurement (%)					
	*N*	<Cutoff	Cutoff + 1 *SD*	Cutoff + 2 *SD*	Cutoff + 3 *SD*	Cutoff + 4 *SD*	*N*	<Cutoff	Cutoff + 1 *SD*	Cutoff + 2 *SD*	Cutoff + 3 *SD*	Cutoff + 4 *SD*
Depression (HSCL-25-D)	27	7.4	11.1	37.0	37.0	7.4	29	27.6	31.0	31.0	10.3	0
Anxiety (HSCL-25-A)	28	14.3	10.7	35.7	35.7	3.6	29	24.1	31.0	31.0	13.8	0
Psychoticism (SCL-90-P)	29	3.4	37.9	24.1	24.1	10.3	27	37.0	37.0	14.8	11.1	0
Somatization (SCL-90-S)	29	20.7	31.0	24.1	20.7	3.4	28	57.1	17.9	14.3	10.7	0
Traumatization (HTQ)	29	31.0	34.5	24.1	6.9	3.4	28	35.7	53.6	10.7	0	0

*HSCL-25-D, Depression subscale of the Hopkins Symptom Checklist; HSCL-25-A, Anxiety subscale of the Hopkins Symptom Checklist; SCL-90-P, Psychoticism subscale of the Symptom Checklist 90-R; SCL-90-S, Somatization subscale of the Symptom Checklist 90-R; HTQ, Traumatization subscale of the Harvard Trauma Questionnaire.*

**Table 4** compares mental health parameters of participants treated in a clinic and participants treated in a PCC. Pre-treatment values did not differ significantly between clinic and PCC, except for pre-treatment depression values being higher in the PCC sample than the clinic sample (*F* = 5.126, *p* < .05).

**Table 4:** Mental health of refugees turning to psychiatric hospitals and PCC (MANOVA).

	Pre-treatment measurement (*N* = 133)		Pre-treatment measurement in clinic (*N* = 54)		Pre-treatment measurement in PCC (*N* = 79)		*F*	*df*	*p*
	*M*	*SD*	*M*	*SD*	*M*	*SD*			
General well-being (WEMWBS)	34.5	(11.9)	36.5	(12.2)	33.1	(11.6)	2.637	1	ns
Depression (HSCL-25-D)	44.3	(8.7)	42.3	(8.9)	45.7	(8.3)	5.126	1	<.05
Anxiety (HSCL-25-A)	28.5	(6.4)	27.8	(6.6)	29.0	(6.3)	1.208	1	ns
Psychoticism (SCL-90-P)	18.1	(10.0)	18.4	(9.8)	17.9	(10.2)	.063	1	ns
Somatization (SCL-90-S)	21.6	(12.6)	22.2	(13.6)	21.3	(12.1)	.176	1	ns
Traumatization (HTQ)	84.3	(18.6)	82.1	(20.7)	85.8	(17.0)	1.334	1	ns
Quality of Life (WHOQoL)	65.8	(15.6)	67.4	(16.8)	64.6	(14.8)	.996	1	ns

*WEMWBS, Warwick Edinburgh Mental Well-Being Scale; HSCL-25-D, Depression subscale of the Hopkins Symptom Checklist; HSCL-25-A, Anxiety subscale of the Hopkins Symptom Checklist; SCL-90-P, Psychoticism subscale of the Symptom Checklist 90-R; SCL-90-S, Somatization subscale of the Symptom Checklist 90-R; HTQ, Traumatization subscale of the Harvard Trauma Questionnaire; WHOQoL, WHO Quality of Life Questionnaire; M, mean; SD, standard deviation; p, level of significance; F, value on the F distribution; test statistics in analysis of variance; df, degrees of freedom; n.s, not significant.*

When comparing post-treatment values to pre-treatment values using a paired *t* test, we found statistically significant improvements in mental health of refugee patients within refuKey in the aggregated sample (clinic and PCC; see **Table 5**). Significantly reduced mental health burden with moderate to high effect sizes (Cohen’s *d* between .5 and 1) was found for the majority of the measured indices including general well-being (*t* = −2.644, *p* < .05, Cohen’s *d* = .499), depression (*t* = 3.902, *p* < .001, Cohen’s *d* = .613), anxiety (*t* = 3.345, *p* < .01, Cohen’s *d* = .751), psychoticism (*t* = 4.945, *p* < .001, Cohen’s *d* = .952), somatization (*t* = 4.807, *p* < .001, Cohen’s *d* = .908), and traumatization (*t* = 2.529, *p* < .05, Cohen’s *d* = .487) but not for quality of life (*t* = −1.816, n.s.) or post-migration stressors (*t* = .919, n.s.). Since post-treatment psychoticism, somatization, and traumatization were not normally distributed and our sample size was slightly smaller than 30 (the minimum sample size for which this violation could be ignored), the Wilcoxon Signed Rank Test was applied additionally, showing the same result (*p* < .001, *p* < .001, *p* < .05, respectively). After Bonferroni correction for multiple testing, the improvements were still significant for depression, anxiety, somatization, and psychoticism.

**Table 5:** Mental health scores of refugee patients before and after treatment within refuKey (paired *t* test).

	*N*	Pre-treatment measurement		Post-treatment measurement		*t*	*df*	*p*	Cohen’s *d*
		*M*	*SD*	*M*	*SD*				
General well-being (WEMWBS)	28	38.5	(15.2)	45.1	(14.6)	−2.644	27	<.05	.499
Depression (HSCL-25-D)	27	41.7	(9.0)	34.2	(11.5)	3.902	26	<.001	.613
Anxiety (HSCL-25-A)	28	27.2	(6.8)	22.8	(7.9)	3.245	27	<.01	.751
Psychoticism (SCL-90-P)	27	21.1	(11.6)	9.2	(8.7)	4.945	26	<.001	.952
Somatization (SCL-90-S)	28	24.9	(14.7)	12.8	(12.9)	4.807	27	<.001	.908
Traumatization (HTQ)	27	79.1	(20.7)	69.2	(19.5)	2.529	26	<.05	.487
Quality of Life (WHOQoL)	25	67.9	(18.8)	74.3	(23.9)	−1.816	24	ns	–
Post-Migration Living Difficulties (PMLDC)	28	58.8	(12.9)	56.0	(15.1)	.919	27	ns	–

*WEMWBS, Warwick Edinburgh Mental Well-Being Scale; HSCL-25-D, Depression subscale of the Hopkins Symptom Checklist; HSCL-25-A, Anxiety subscale of the Hopkins Symptom Checklist; SCL-90-P, Psychoticism subscale of the Symptom Checklist 90-R; SCL-90-S, Somatization subscale of the Symptom Checklist 90-R; HTQ, Traumatization subscale of the Harvard Trauma Questionnaire; WHOQoL, WHO Quality of Life Questionnaire; PMLDC, Post-Migration Living Difficulties Checklist; M, mean; SD, standard deviation; p, level of significance; t, value on the t distribution; test statistics in a t test; df, degrees of freedom; Cohen’s d, measure of effect size; n.s, not significant.*

Post-migration living difficulties reported by participants are shown in **Table 6**. Family issues (separation from family, worries about family back home, inability to return to home country in case of emergency, loneliness), asylum procedure (fear of being sent back to country of origin), and socioeconomic living conditions (difficulties with employment, difficulties obtaining appropriate accommodation) represented major problems for the participants.

**Table 6:** Prevalence of post-migration living difficulties (PMLDC) before treatment.

	*N*	*M*	*SD*
Communication difficulties	134	3.18	(1.31)
Discrimination	125	2.64	(1.45)
Conflicts with your own/other ethnic groups in Germany	128	1.94	(1.42)
Separation from your family	130	3.68	(1.51)
Worries about family back home	131	4.02	(1.37)
Being unable to return to your home country in an emergency	128	4.09	(1.46)
Difficulties with employment (being permitted to work, finding work, bad working conditions, etc.)	123	3.53	(1.50)
Difficulties in interviews with immigration officials	131	3.22	(1.67)
Conflicts with social workers/other authorities	128	1.95	(1.42)
Not being recognized as a refugee	119	3.35	(1.76)
Being fearful of being sent back to your country of origin in the future	132	4.63	(.96)
Worries about not getting access to treatment for health problems	133	3.38	(1.57)
Not enough money to buy food, pay the rent, or buy necessary clothes	131	3.11	(1.46)
Difficulties obtaining financial assistance	124	2.79	(1.58)
Loneliness, boredom, or isolation	131	4.15	(1.23)
Difficulties learning German	133	3.26	(1.41)
Difficulties obtaining appropriate accommodation	130	3.76	(1.50)

Finally, **Table 7** presents correlations of the Post-Migration Living Difficulties index with the measured mental health indices. Each of these correlations is both highly significant as well as moderate in strength ranging from *r* = −.250 for general well-being to *r* = −.537 for quality of life.

**Table 7:** Correlation between mental health indices and Post-Migration Living Difficulties Scale (PMLDC, Pearson’s correlation analysis).

	*N*	*r*	*Sig.* (2-tailed)
General well-being (WEMWBS)	131	−.250**	.004
Depression (HSCL-25-D)	129	.415**	.000
Anxiety (HSCL-25-A)	131	.341**	.000
Psychoticism (SCL-90-P)	133	.367**	.000
Somatization (SCL-90-S)	134	.401**	.000
Traumatization (HTQ)	130	.457**	.000
Quality of Life (WHOQoL)	132	−.537**	.000

*WEMWBS, Warwick Edinburgh Mental Well-Being Scale; HSCL-25-D, Depression subscale of the Hopkins Symptom Checklist; HSCL-25-A, Anxiety subscale of the Hopkins Symptom Checklist; SCL-90-P, Psychoticism subscale of the Symptom Checklist 90-R; SCL-90-S, Somatization subscale of the Symptom Checklist 90-R; HTQ, Traumatization subscale of the Harvard Trauma Questionnaire; WHOQoL, WHO Quality of Life Questionnaire; PMLDC, Post-Migration Living Difficulties Checklist; r Pearson’s correlation coefficient, Sig. level of significance.*

## Discussion

The present paper describes the implementation and scientific evaluation of the refuKey project in Lower Saxony, Germany. RefuKey aims to reduce cultural and structural barriers to mental health care faced by refugees by using a need-adapted stepped-care approach and to promote intercultural opening in psychiatric routine care by using a multi-centric approach. The refuKey project offers a differentiated support and care model that includes psychiatric and psychotherapeutic treatments as well as psychosocial counseling with the involvement of interpreters, giving refugees low-threshold access to mental health care services. Furthermore, it provides qualified training in transcultural competence, interpreter-assisted psychotherapy, bureaucratic and legal issues, etc. for mental health care professionals and interpreters. With this approach, refuKey incorporates into its mental health care model all the aspects subsequently recommended in a position paper by the National Academy of Science in Germany (37).

Evidence on refugee mental health care in psychiatric settings in Germany is generally quite scarce. Our secondary data survey provides first insight into the current situation of psychiatric-psychotherapeutic routine care for refugees in Lower Saxony, Germany. The survey shows large differences in the numbers of treated refugees between the psychiatrics clinics as reported by chief physicians, suggesting different levels of transcultural opening in these mental health institutions. Another important finding is the lack of systematic documentation of refugees in psychiatric clinics, explaining the low rate of return in our survey. Records about the authority covering the costs of treatment are often the only indication of refugee status of a patient. Furthermore, the authorities responsible for refugees’ health care differ between federal states and change depending on the duration of stay^5^. Due to these differences, some of the refugees cannot be identified as such in the documentation systems of clinics, which makes it difficult to make inferences about the mental health care of this vulnerable group in Germany. An attempt to standardize the documentation processes in medical institutions in Germany is therefore indispensable for improving intercultural opening and finding new strategies for refugee mental health care.

Additionally, in the secondary data collection, chief physicians of psychiatric clinics reported various barriers with a negative impact on the quality of mental health care of refugees. The most commonly reported barriers were 1) communication problems due to lack of language proficiency and 2) the resulting additional time required for psychiatric assessment. Experts also pointed out several difficulties concerning their work with refugees. These included health care barriers (e.g., insecure residence status, bureaucratic burden, linguistic problems) as well as difficulties associated with characteristics of the refugee population itself (e.g., lack of culturally sensitive treatment options, culturally shaped disease patterns, refugees’ living conditions). These findings are in line with various structural and social barriers faced by refugees in the health care system that are reported in the literature (15). They also highlight the need for an intercultural opening of the health care system that would, for example, include culturally adapted interventions, cultural competence training for mental health care professionals as well as availability of professional interpreters to overcome language barriers, as implemented by the refuKey project.

First preliminary findings on the treatment of refugees within refuKey showed significant improvements (both statistically and in terms of effect sizes) on most general and symptom-specific outcome measures. RefuKey patients displayed increased general well-being and lower depression, anxiety, psychoticism, somatization, and traumatization values at the end of treatment, while perceived quality of life did not increase significantly, which could be ascribed to insufficient statistical power due to small sample size. As the post-migration stressors did not change over the course of the treatment and despite the fact of possible confounders, the improvement in refuKey patients’ mental health might be attributable to the treatment itself. However, a control group is needed to evaluate the specific effectiveness of treatment within refuKey as compared to treatment as usual.

Another important point to emphasize is that refugees admitted to psychosocial counseling centers showed psychiatric symptom severity levels similar to those admitted to psychiatric clinics. In this context, it is especially important to distinguish between the refugees who could be treated solely by psychosocial interventions in the PCCs and those who display more severe symptoms, in order to provide sufficient care for the latter group through cooperation and networking between PCCs and psychiatric clinics. This finding speaks in favor of a need-adapted stepped-care model with cooperating competence centers as implemented in the refuKey project to overcome structural and social barriers. In this sense, experience that will be gained in the course of the refuKey project regarding the close collaboration between PCCs and psychiatric clinics will deepen our understanding of how to implement stepped-care approaches, especially concerning the extent of the needed support and the question of how to overcome access barriers.

Additionally, we examined the relationship between post-migratory living difficulties and indicators of mental health in refugees treated within refuKey and found an association between the two. Specifically, a higher load of post-migration stressors was strongly associated with lower quality of life and moderately associated with lower general well-being. A moderate association of post-migratory living difficulties with more severe syndrome-specific outcomes such as symptoms of depression, anxiety, psychoticism, somatization, and traumatization was similarly observed. Exposure to multiple post-migration stressors combined with psychological resource constraints faced by refugees may lead to poorer mental health and thus explain this association(38). In this study, the main stressors reported by refugee participants were fear of deportation, concerns about family members back home, inability to return home in case of emergency as well as loneliness, boredom, and isolation. Similarly, studies examining the nature of post-migration stressors in samples of asylum seekers reported fear of deportation and concerns about family members back home, but also delays in processing asylum applications, and not having a work permit as main stressors (39, 40). Furthermore, the link between psychoticism and post-migration stressors has been addressed by research showing elevated risk of psychosis not only in first- but also in second-generation migrants (6). Selten and Cantor-Graae, (41) suggest that social defeat, defined as the long-term experience of an outsider status or a subordinate position, might be the mechanism underlying the enhanced risk of psychosis in the respective population.

Interestingly, we found no increase in quality of life after treatment but observed a strong negative association between quality of life and post-migration stressors. This finding might suggest that in contrast to the other mental health measures, quality of life depends more on factors such as post-migration stressors, which are not solely influenced by a psychotherapeutic or psychiatric treatment. Even though our analysis of the former relationship does not allow causal inferences, it seems likely that attempts to reduce post-migration stressors could improve quality of life in refugees. This hypothesis is supported by longitudinal evidence examining distress in refugees in relation to the state of their asylum-seeking process, which revealed a decrease in mental health burden only for those refugees who obtained a positive legal status outcome within the measurement data points (40). Moreover, Laban, Gernaat, Komproe, Schreuders, and De Jong (42) compared recently arrived (<6 months) with long-term (>2 years) asylum seekers and found increased prevalence of psychiatric disorders in the long-term group, which implies an influence of post-migration factors on mental health. Thus, it would be beneficial to the mental health of refugees to reduce post-migration stressors through appropriate administrative changes to the asylum procedure and social policies. Providing culturally adapted and timely mental health care for refugees including support for social integration has also positive socio-political consequences. Integration of refugees to the host country is a manifold and multifaceted process, including adaptation into the educational, labor, health, and community systems and affected by various factors such as pre- and post-migratory experiences and openness of the host society towards cultural diversity (43). Moreover, it has been shown that the psychiatric impairment goes along with poor integration measures in refugees (35). Therefore, early recognition and mitigation of the psychiatric symptoms *via* culture-sensitive and need-adapted approaches are crucial to promote social integration and psycho-social functioning of the refugees who have to contend not only with their previous traumatic experiences but also with the post-migratory stress factors.

With our primary dataset, we provide a naturalistic overview of various demographic and clinical variables in a help-seeking refugee population. Therefore, our results should not be generalized to refugees outside psychiatric and psychosocial settings. Furthermore, no causal conclusions regarding post-migration factors can be drawn from our data, since it is cross-sectional. Additionally, since this is an ongoing study, the statistical power is still low. Therefore, we could not yet control for covariates within our analysis and cannot exclude that the results are influenced by a third variable (e.g., country of origin, changes in asylum status), so that the results should be interpreted with caution. Self-reports were provided in the patients’ native language to overcome a possible source of bias, which can be regarded as one of the key strengths of our study. It is also important to emphasize that it is challenging to assess the effectiveness of these complex interventions in a way that allows for drawing robust conclusions. For that reason, further evaluation strategies such as prospective comparative methods will be combined with the ongoing evaluation process.

To conclude, this study provides the first scientific naturalistic preliminary dataset from the implementation of an innovative need-adapted stepped-care model project (refuKey) in Lower Saxony, Germany. The existence of structural and social barriers regarding the access of refugees to adequate and high-quality mental health care was discussed. Our data also support findings concerning the impact of the post-migratory living difficulties on mental health and quality of life of refugees. In addition, the need for stepped-care approaches and firm networking between psychosocial centers and psychiatric clinics was underlined. First results suggest a positive impact of the project on the mental health care situation of asylum seekers who seek help from refuKey mental health care services and indicate that the mental health care model implemented by the project might serve as a good adaptation to the needs of refugees.

## Data Availability

The datasets generated for this study are available on request to the corresponding author.

## Ethics Statement

The studies involving human participants were reviewed and approved by Ethics committee of the Hanover Medical School. The patients/participants provided their written informed consent to participate in this study.

## Author Contributions

BT, LE, IO, and IG-C contributed conception and design of the study. BT, LE, KL, and GP contributed to the acquisition of data. LE organized the database. BT performed the statistical analysis. BT, UA, and JW wrote sections of the manuscript. All authors contributed to manuscript revision, read and approved the submitted version.

## Funding

The project and the research for this paper were financially supported by the Ministry of Social Affairs, Health and Equality of Lower Saxony (grant number: 4 SL 1.6.-41583/2017-8).

## Conflict of Interest Statement

The authors declare that the research was conducted in the absence of any commercial or financial relationships that could be construed as a potential conflict of interest.

## Footnotes

1 In this article, we prefer to use the term “refugees” as an umbrella term, which denotes all asylum seekers regardless of the recognition of their claim.

2 Asylum Seekers Benefits Act.

3 Psychiatry conference Lower Saxony (Niedersächsische Psychiatrie Konferenz).

4 GPower analysis indicated a 93% chance of detecting a large effect size and a 49% chance of detecting a medium effect size (G*Power uses f2 as the effect size parameter (.12 = .01 is small, .252 = .0625 is medium, and .402 = .16 is large) between the two groups as significant at the 5% level (two tailed).

5 Within the first 15 months, the authorities determined by federal law are responsible for the provision of health care services for refugees (§4&6 AsylbLG). Afterwards, refugees receive the benefits of health insurance (§2 AsylbLG, §264 SGB V) that are covered by the social welfare office.
